# Supplementary material for: Unraveling the molecular mechanism of MIL-53(Al) crystallization
Source: Nat Commun. 2022 Jun 29;13:3762. doi: 10.1038/s41467-022-31294-4 (PMC9243051; doi:10.1038/s41467-022-31294-4)
Supplement: Supplementary file 1 — Supplementary Info [file 41467_2022_31294_MOESM1_ESM.pdf]

Supporting Information for

Unraveling the Molecular Mechanism of MIL-53(Al) Crystallization

Daniil Salionov[a], Olesya O. Semivrazhskaya[b], Nicola P. M. Casati[c], Marco Ranocchiari[d], Saša Bjelić,[a] René Verel[e], Jeroen A. van Bokhoven[d,e,*], Vitaly L. Sushkevich[d,*]

[a] *Bioenergy and Catalysis Laboratory, Paul Scherrer Institute, 5232 Villigen PSI, Switzerland*

[b] *Laboratory for Organic Chemistry, ETH Zürich, Vladimir-Prelog-Weg 3, 8093 Zürich, Switzerland*

[c] *Laboratory for Synchrotron Radiation - Condensed Matter, Paul Scherrer Institute,5232 Villigen PSI, Switzerland*

[d] *Laboratory for Catalysis and Sustainable Chemistry, Paul Scherrer Institute, 5232 Villigen PSI, Switzerland*

[e] *Institute for Chemistry and Bioengineering, ETH Zurich, Vladimir-Prelog-Weg 1, 8093 Zurich, Switzerland*

Supplementary discussion

Isotope tracing for denoting HRMS peaks

Diversity of chemical reactions and the presence of several ligands in the reaction mixture does not allow deciphering of the entire spectrum; however, using isotope tracing, we were able to assign the majority of the most intense peaks. Apart from the reaction of the non-labelled reagents, four experiments with labelled compounds were carried out, namely

- $^{13}C_2$-labelled terephthalic acid with two ^{13}C atoms in carboxyl position
- Fully deuterated N,N-dimethylformamide-d_7.
- Fully deuterated methanol-d_4, used as the solvent for sample dilution prior to the injection to the HRMS machine
- ^{15}N-labelled aluminum nitrate hydrate

The synthetic composition employed in the isotope tracing experiment was identical to the one used in unlabeled experiments, in particular, $c(Al(NO_3)_3)$ = 0.66 M and

$c(H_2BDC)$ = 0.33 M. Reaction temperature was about 353 K. Experiments with methanol were performed due to the apparent interaction of OH groups of secondary building units with methanol upon dilution, leading to methoxy species, which presence required experimental confirmation and quantification.

Supplemantary Tables 1 and 2 report the most intense peaks observed in HRMS experiments at different reaction times together with their assignment. The calculated and measured masses are given for comparison; the absolute accuracy in most of the cases was better than 20 ppm, typical for ESI Q-TOF experiments. Some peaks had worse precision, and their assignment might require further clarification. Relative isotope shift observed upon isotope substitution is provided in integer values for the sake of clarity. Supplementary Figures 1-4 report the exemplary HRMS spectra for the corresponding isotope tracing experiments. Three repetitive test under same conditions showed full reproducibility of the presented data, either isotope shifts or temporal behavior.

Time-resolved MAS NMR measurements

To enable high time resolution, in all experiments $^{13}C_2$-labelled terephthalic acid with two ^{13}C atoms in carboxyl position was used. The processed superimposed MAS NMR spectra used for quantitative analysis are presented in Supplementaly Figures 5-16. For careful assignment of the signals in ^{1}H NMR, the experiment in DMF-d_7 was carried out (Figure 20). To follow the fate of formate species formed during hydrolysis of DMF, the NMR experiment with ^{13}C-DMF was carried out, with ^{13}C atom located in formate group; methyl groups were left unlabeled.

Due to the spinning at magic angle with considerable speed (above 2.8 kHz), liquids, solids and suspensions can undergo spontaneous heating due to viscous friction. Moreover, the measured temperature of the bearing gas inside NMR probe can be different from the temperature inside the stator, rotor and synthetic mixture. Therefore, the temperature calibration inside the reaction ampule is essential. We used standard approach used in liquid-phase NMR, implying the utilization of 80% ethylene glycol in d_6-DMSO mixture, which is commonly used as NMR-thermometer. This mixture was sealed in NMR inserts and spun under magic angle while heated at different

temperatures. ^{1}H single pulse spectra (4 scans, recycling delay 10 s) were recorded to determine the chemical shifts of CH_2 and OH groups. The difference in their position was used to calculate the actual temperature of the liquid in the rotor under magic angle spinning. The corresponding calibrated temperatures were used for the selection of conditions in MOF synthesis experiments.

Several repetitive tests carried out at 343 K showed complete reproducivility of kinetic profiles reported in the main text. Variation of the mixing time during CP-MAS experiemnts from 1000 to 5000 μs revealed no effect on the detection sensitivity and the shape of kinetic profiles.

Supplementary Figures

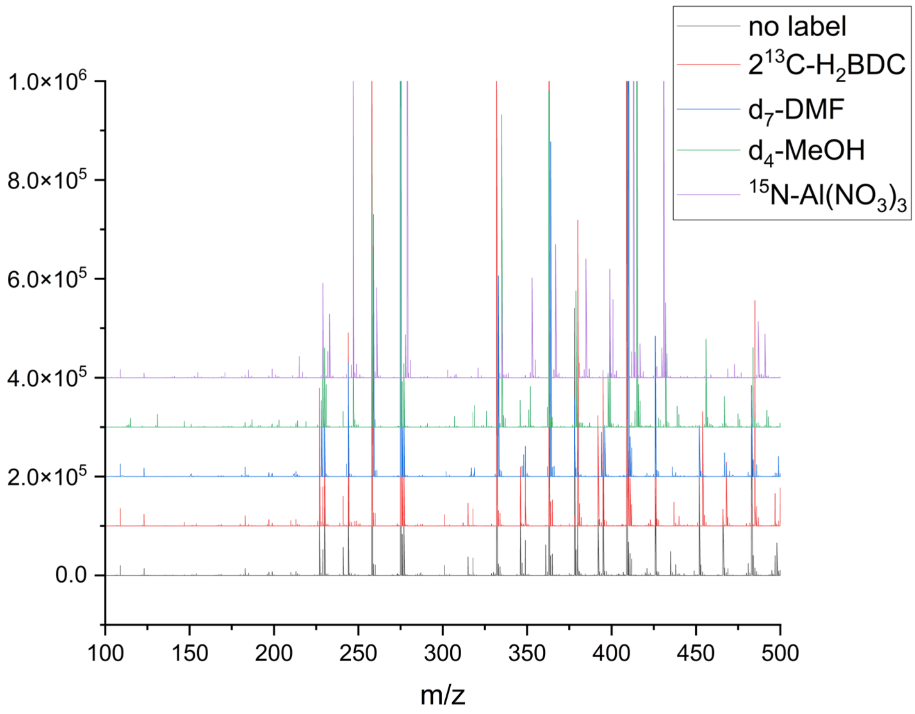

Supplementary Figure 1. Negative mode ESI Q-TOF spectra of the synthetic mixture prepared using different isotopically labeled compounds compared to the unlabeled in the range of 100-500 m/z. Reaction time 200±20 min, temperature 353±2 K.

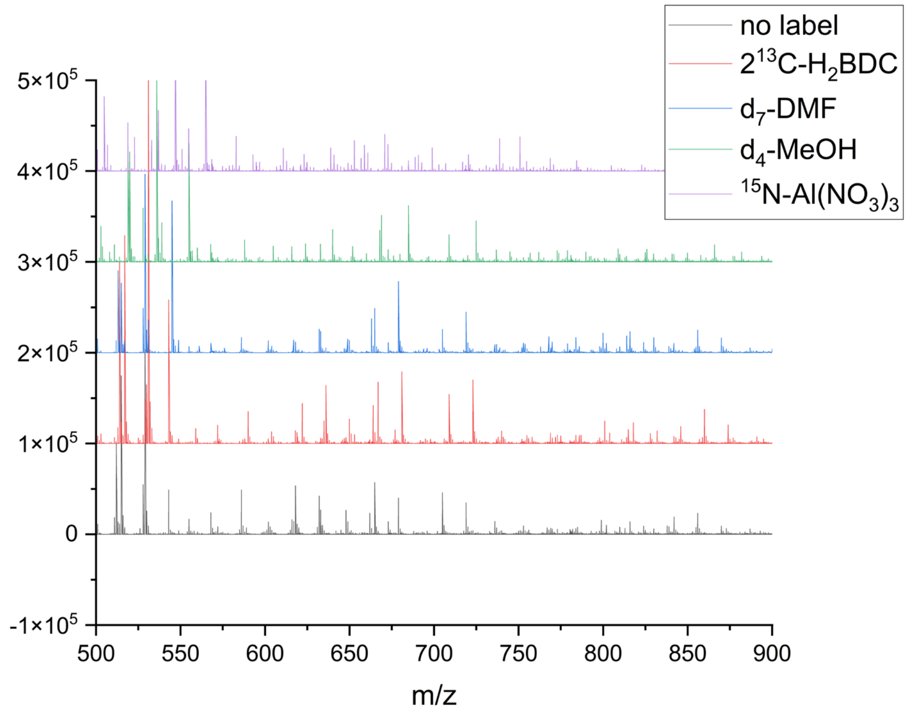

Supplementary Figure 2. Negative mode ESI Q-TOF spectra of the synthetic mixture prepared using different isotopically labeled compounds compared to the unlabeled in the range of 500-900 m/z. Reaction time 200±20 min, temperature 353±2 K.

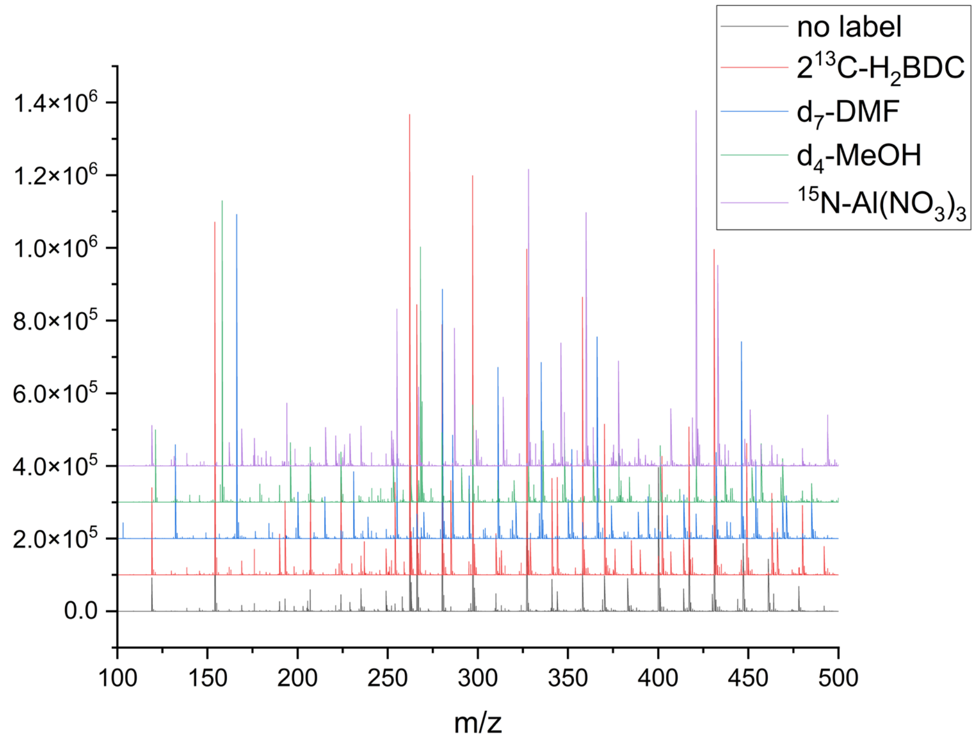

Supplementary Figure 3. Positive mode ESI Q-TOF spectra of the synthetic mixture prepared using different isotopically labelled compounds compared to the unlabeled in the range of 100-500 m/z. Reaction time 200±20 min, temperature 353±2 K.

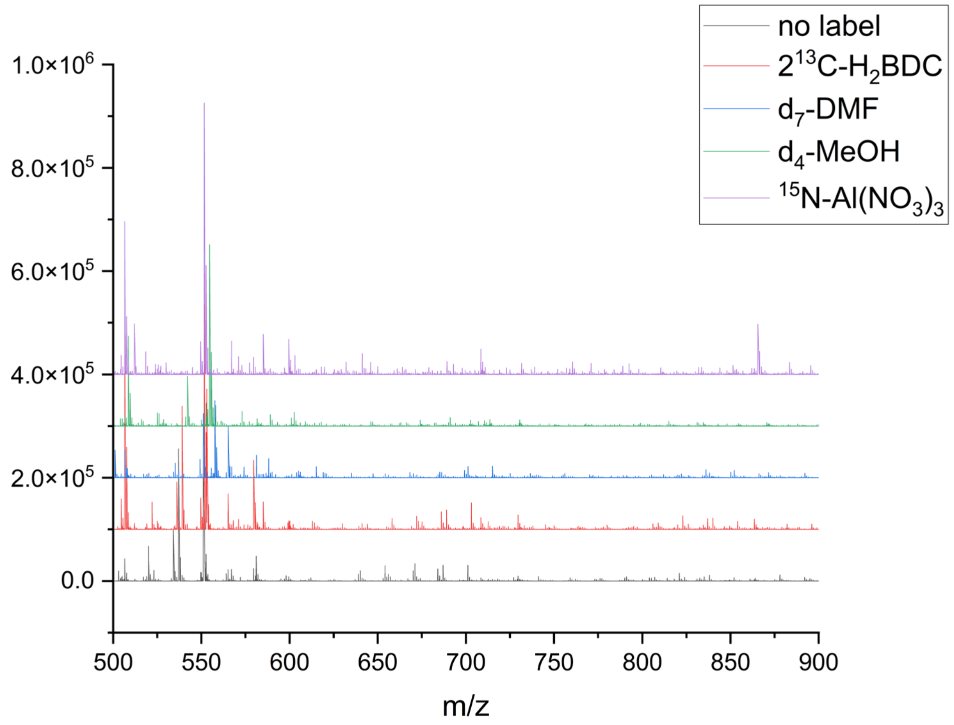

Supplementary Figure 4. Positive mode ESI Q-TOF spectra of the synthetic mixture prepared using different isotopically labelled compounds compared to the unlabeled in the range of 500-900 m/z. Reaction time 200±20 min, temperature 353±2 K.

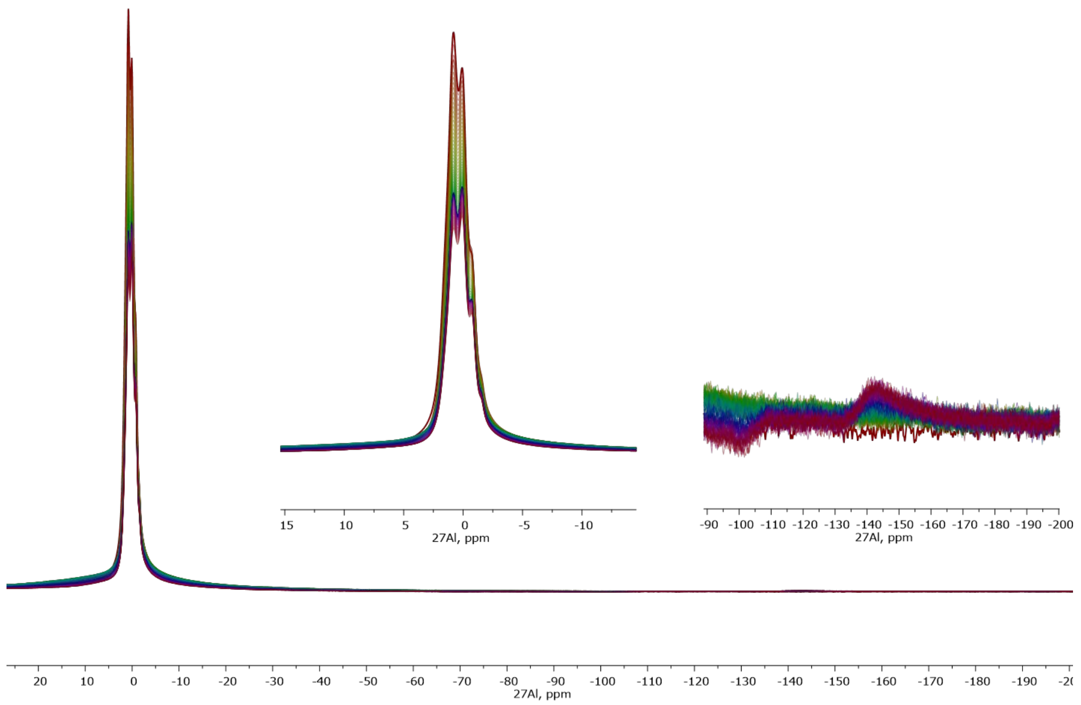

Supplementary Figure 5. Time-resolved ^{27}Al MAS NMR spectra acquired during the synthesis of MIL-53 at 333 K.

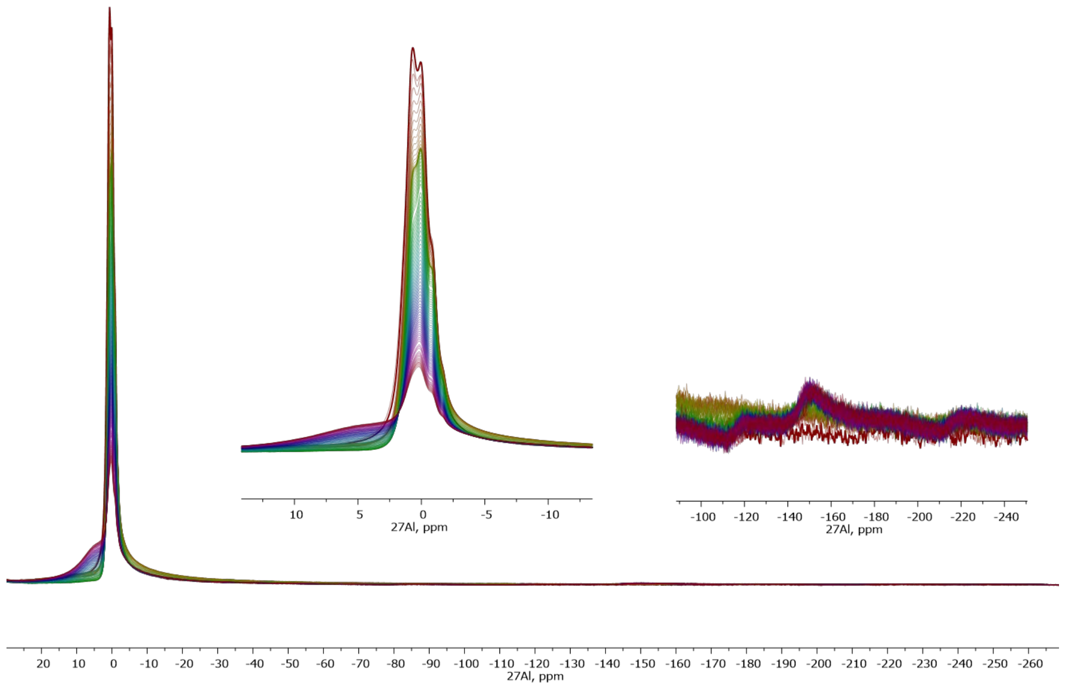

Supplementary Figure 6. Time-resolved ^{27}Al MAS NMR spectra acquired during the synthesis of MIL-53 at 343 K.

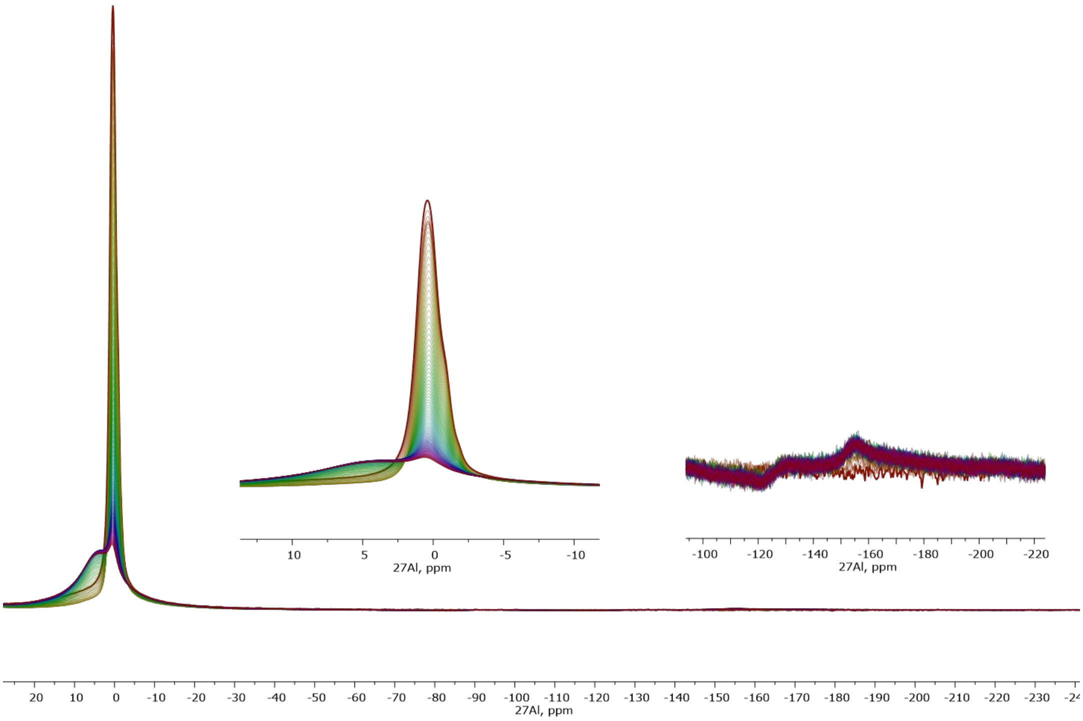

Supplementary Figure 7. Time-resolved ^{27}Al MAS NMR spectra acquired during the synthesis of MIL-53 at 353 K.

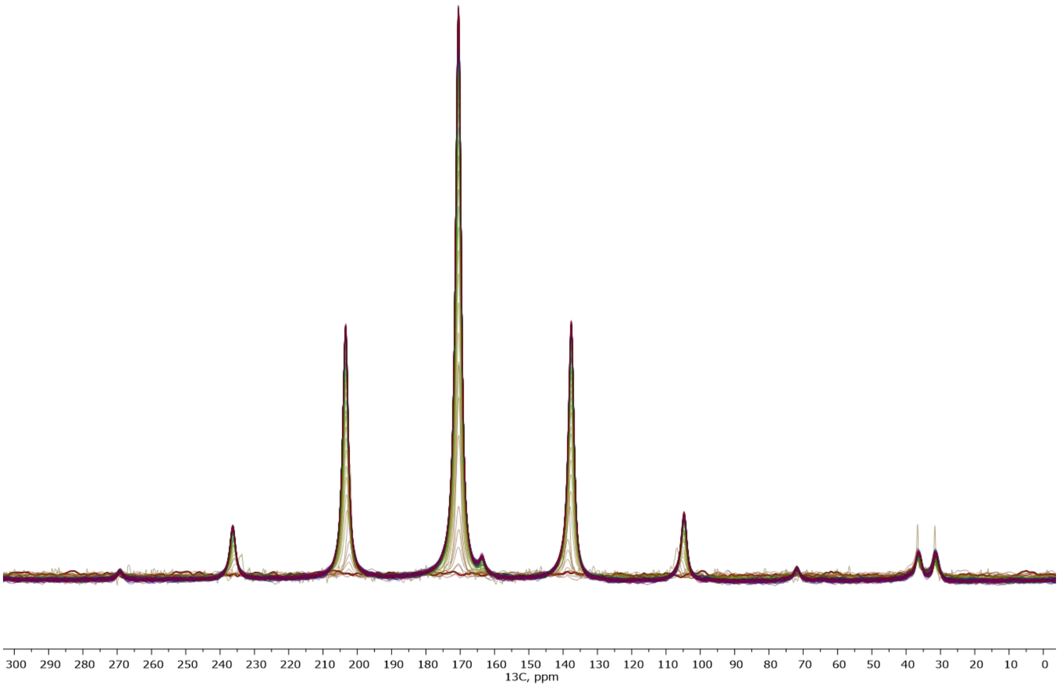

Supplementary Figure 8. Time-resolved ^{13}C CP MAS NMR spectra with proton decoupling acquired during the synthesis of MIL-53 at 333 K.

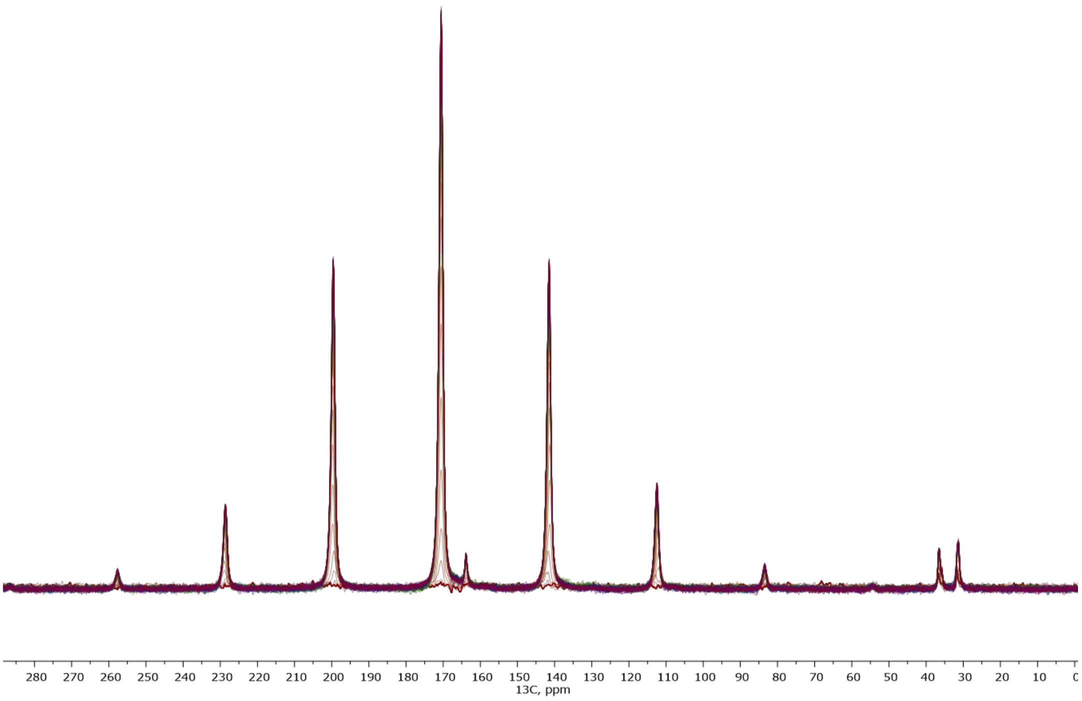

Supplementary Figure 9. Time-resolved ^{13}C CP MAS NMR spectra with proton decoupling acquired during the synthesis of MIL-53 at 343 K.

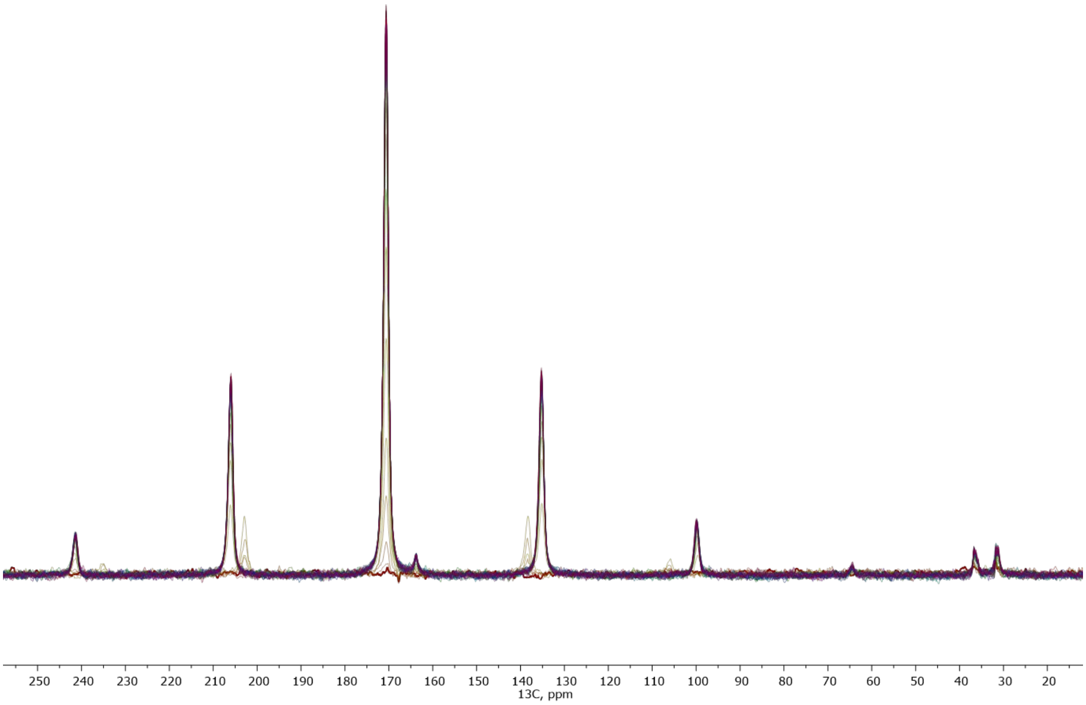

Supplementary Figure 10. Time-resolved ^{13}C CP MAS NMR spectra with proton decoupling acquired during the synthesis of MIL-53 at 353 K.

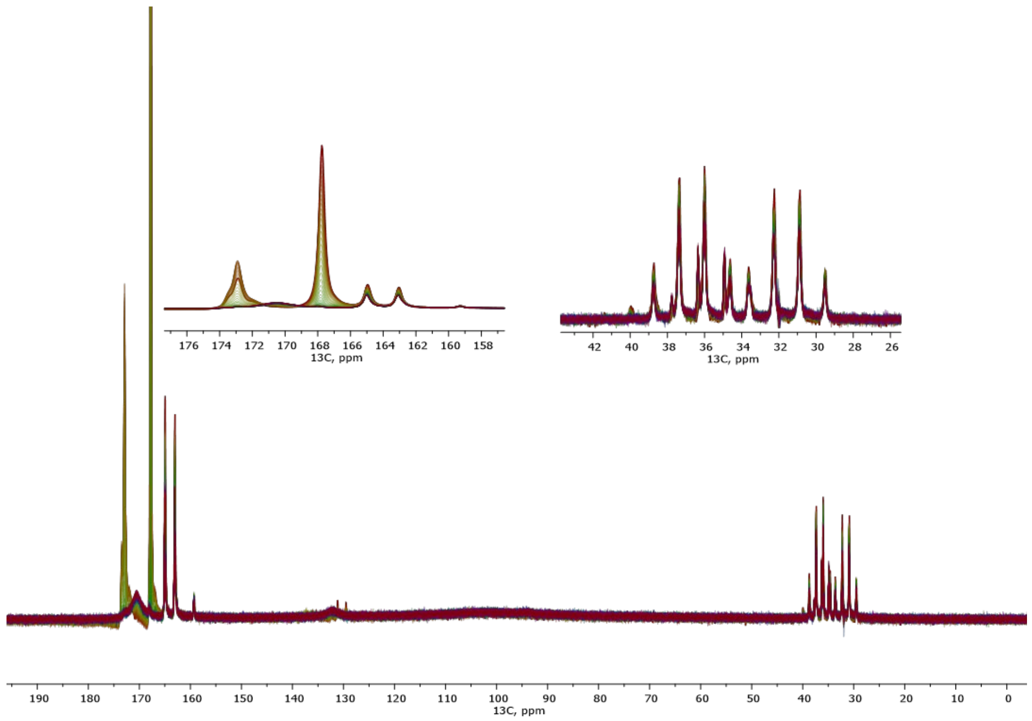

Supplementary Figure 11. Time-resolved ^{13}C DE MAS NMR spectra without proton decoupling acquired during the synthesis of MIL-53 at 333 K.

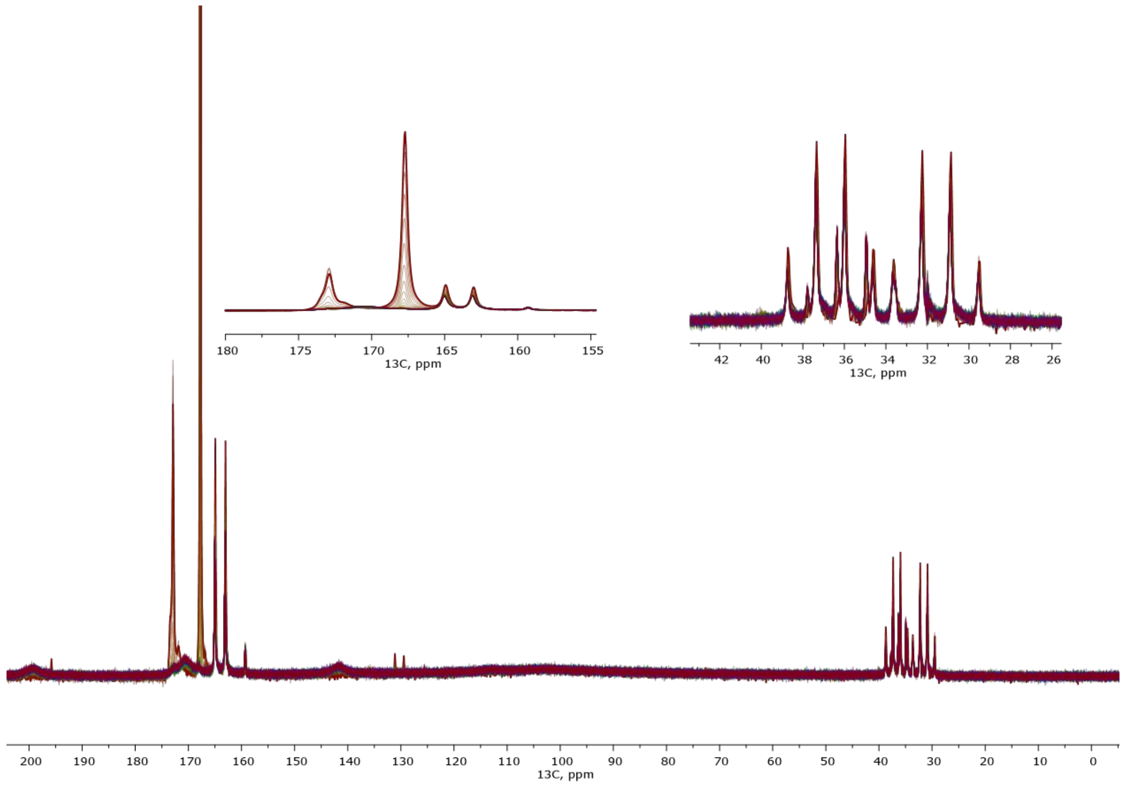

Supplementary Figure 12. Time-resolved ^{13}C DE MAS NMR spectra without proton decoupling acquired during the synthesis of MIL-53 at 343 K.

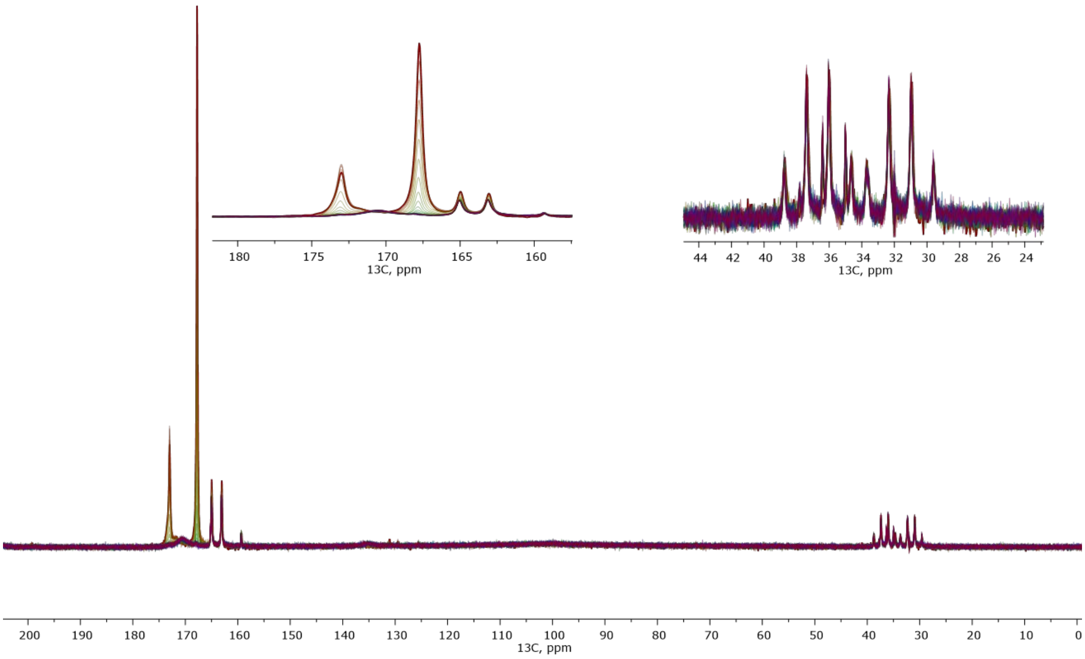

Supplementary Figure 13. Time-resolved ^{13}C DE MAS NMR spectra without proton decoupling acquired during the synthesis of MIL-53 at 353 K.

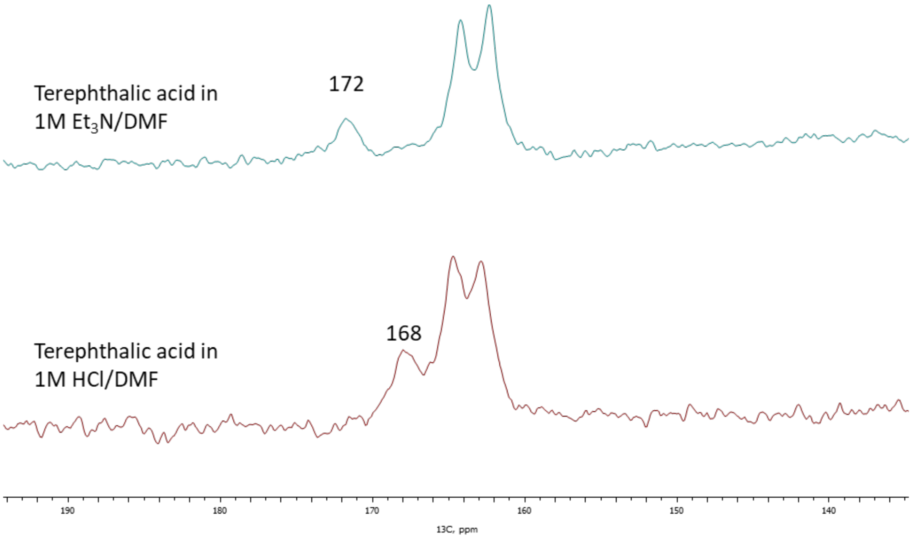

Supplementary Figure 14. ^{13}C NMR spectra of terephthalic acid in 1M HCl and Et_3N solutions in DMF. These two solutions mimic the acidic and basic media, shifting the protonation-deprotonation equilibrium for terephthalic acid. In HCl/DMF mixture terephthalic acid will be fully protonated, while in Et_3N/DMF it should be completely deprotonated. The corresponding spectra show that protonated terephthalate possesses the signal at 172 ppm, while deprotonated has the signal at 168 ppm.

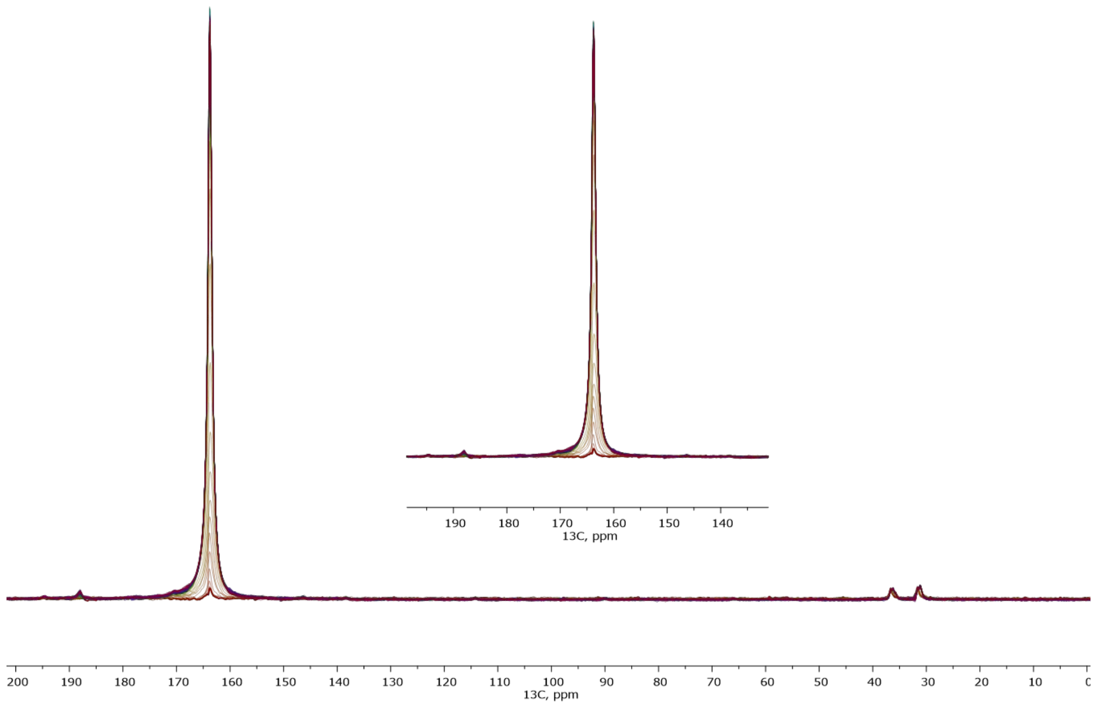

Supplementary Figure 15. Time-resolved ^{13}C CP MAS NMR spectra with proton decoupling acquired during the synthesis of MIL-53 at 343 K in ^{13}C-DMF.

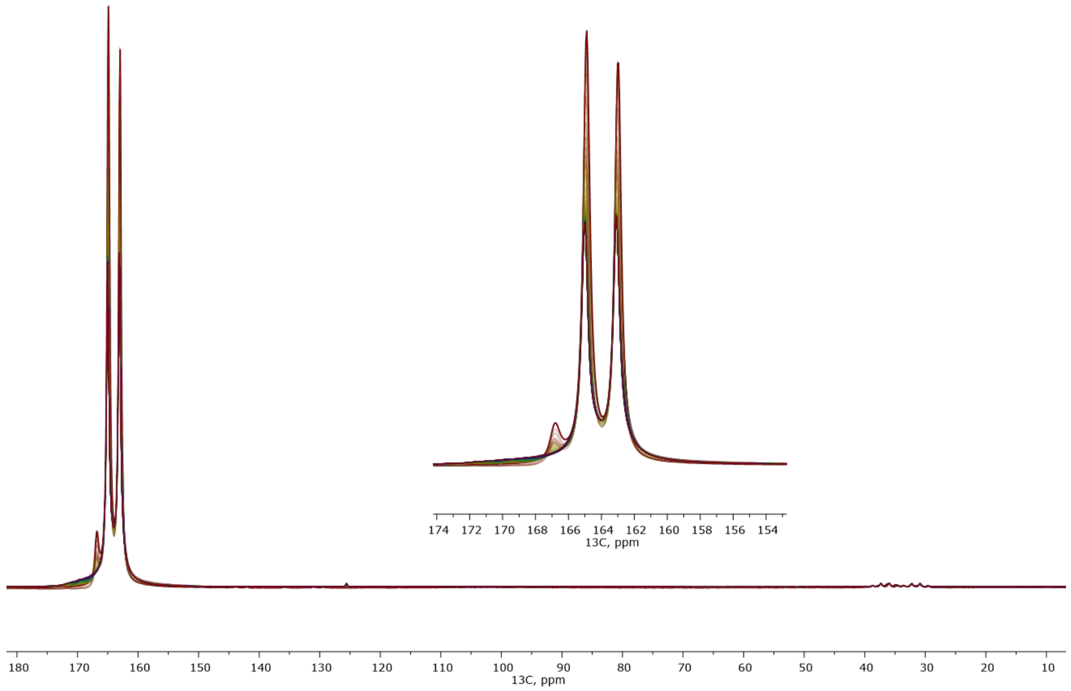

Supplementary Figure 16. Time-resolved ^{13}C DE MAS NMR spectra without proton decoupling acquired during the synthesis of MIL-53 at 343 K in ^{13}C-DMF.

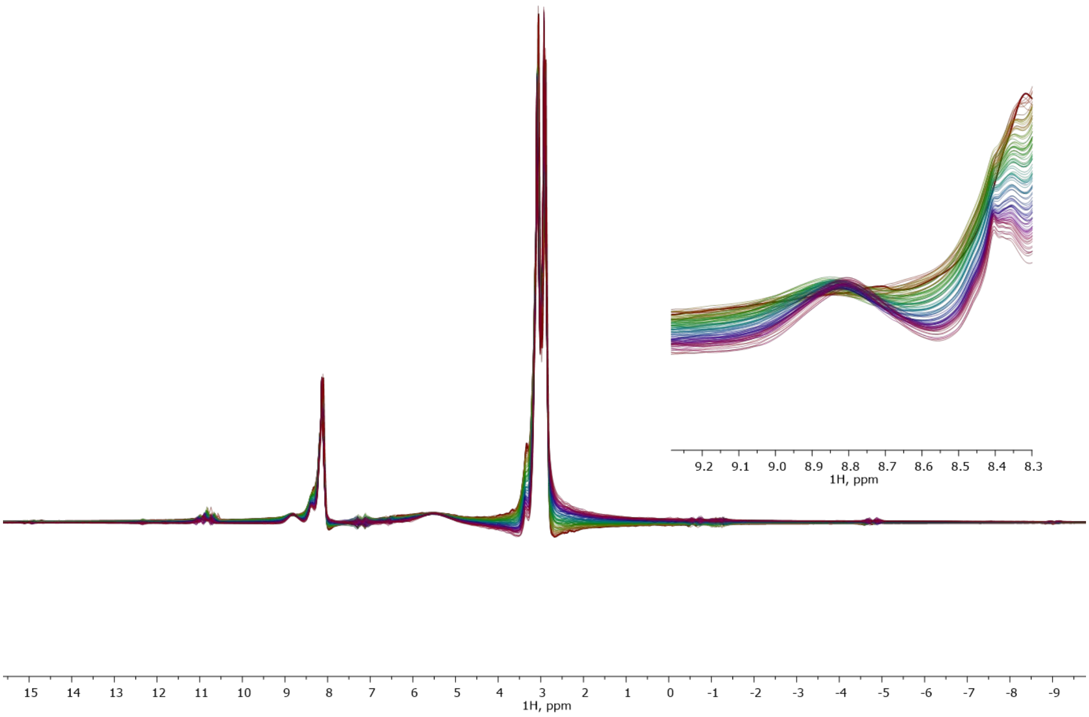

Supplementary Figure 17. Time-resolved ^{1}H MAS NMR spectra acquired during the synthesis of MIL-53 at 333 K.

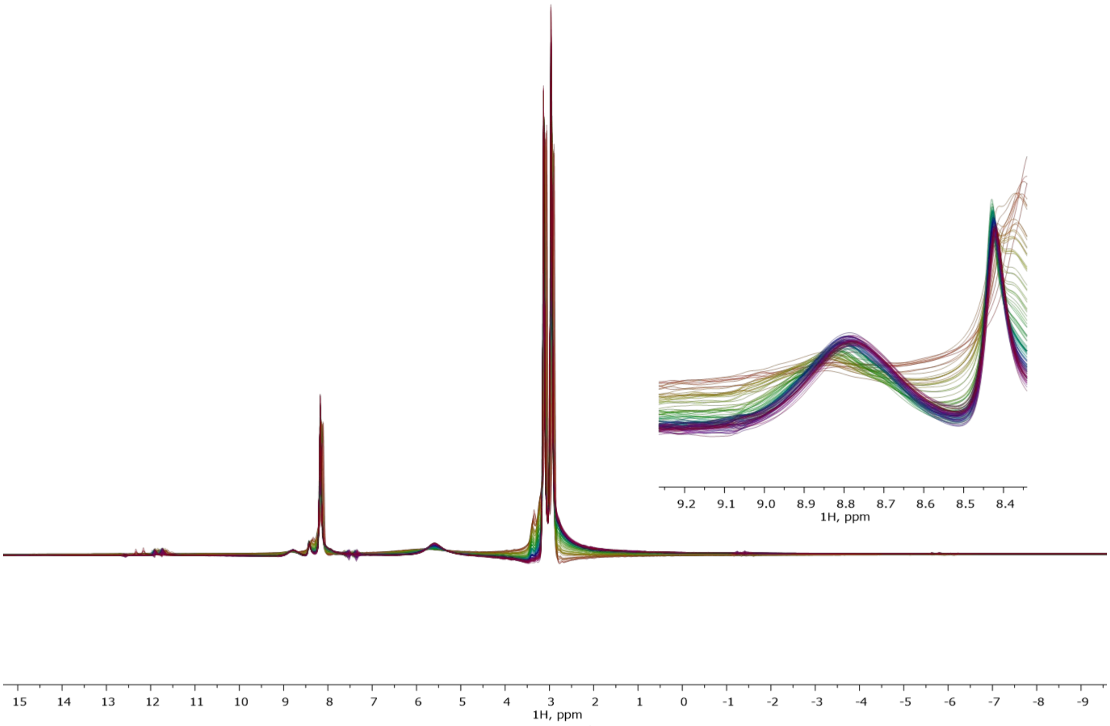

Supplementary Figure 18. Time-resolved ^{1}H MAS NMR spectra acquired during the synthesis of MIL-53 at 343 K.

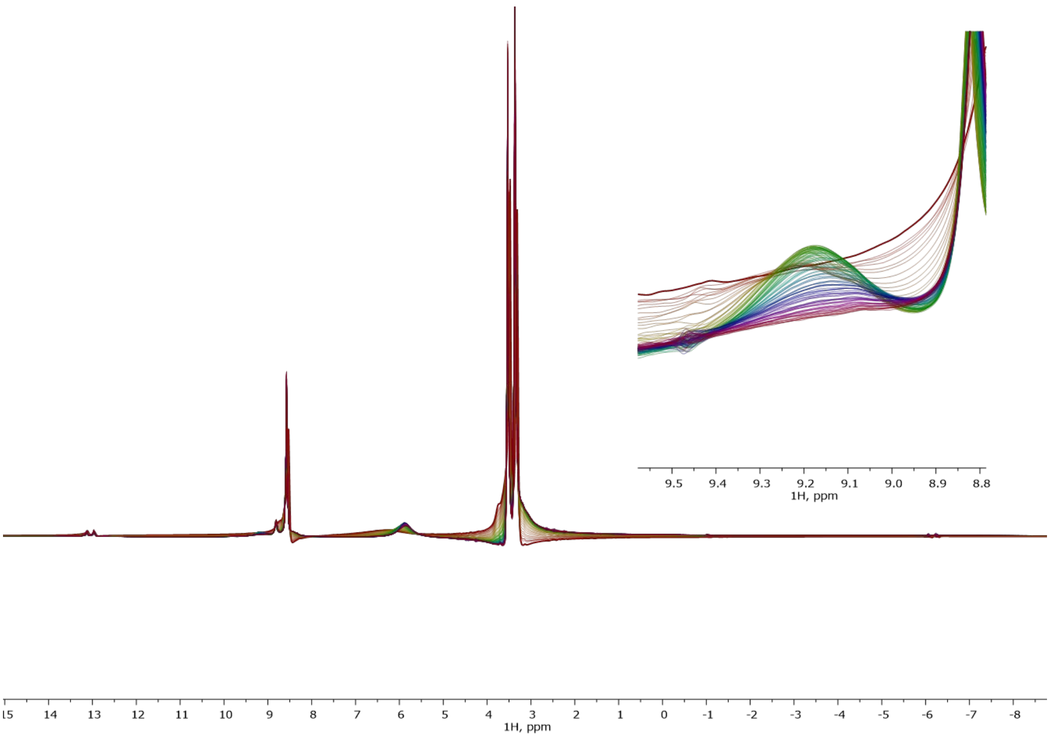

Supplementary Figure 19. Time-resolved ^{1}H MAS NMR spectra acquired during the synthesis of MIL-53 at 353 K.

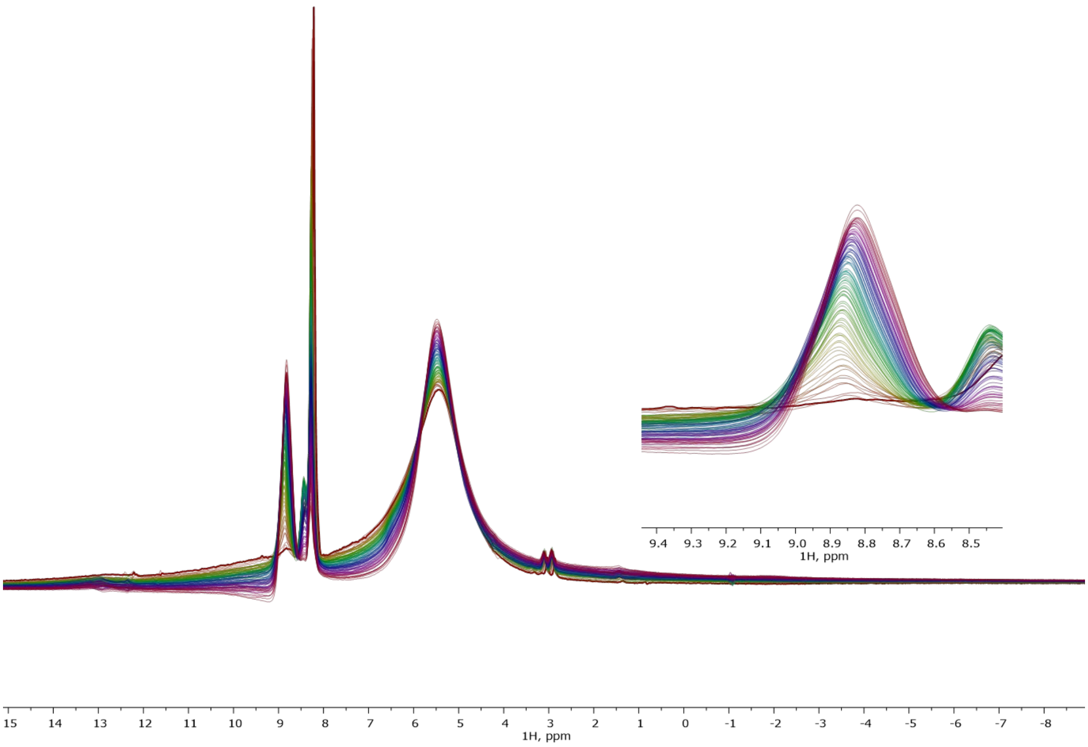

Supplementary Figure 20. Time-resolved ^{1}H MAS NMR spectra acquired during the synthesis of MIL-53 at 333 K in d_7-DMF.

Supplementary Tables

Supplementary Table 1. Measured and calculated m/z as well as their assignment in negative mode.

m/z observed	m/z calculated	Error, ppm	Assigned to	Δ m/z in terephthalic-$^{13}C_2$ acid	Δ m/z in DMF-d_7	Δ m/z in methanol-d_4	Δ m/z in ^{15}N aluminum nitrate
109.0068	109.0087	17	$Al(OCH_3)(OH)_3^-$	0	0	+6	0
123.0226	123.0243	14	$Al(OCH_3)_2(OH)_2^-$	0	0	+8	0
226.9714	226.9737	10	$Al(NO_3)_2(OCH_3)(HCOO)^-$	0	+1	+3	+2
229.9461	229.9482	9	$Al(NO_3)_3(OH)^-$	0	0	+1	+3
243.9615	243.9639	10	$Al(NO_3)_3(OCH_3)^-$	0	0	+3	+3
257.9408	257.9431	9	$Al(NO_3)_3(HCOO)^-$	0	+1	0	+3
274.9320	274.9333	5	$Al(NO_3)_4^-$	0	0	0	+4
331.9347	331.9380	10	$Al_2O(NO_3)_3(OCH_3)(HCOO)^-$	0	+1	+3	+3
345.9112	345.9173	18	$Al_2O(NO_3)_3(HCOO)_2^-$	0	+2	0	+3
348.9246	348.9282	10	$Al_2O(NO_3)_4(OCH_3)^-$	0	0	+3	+4
362.9038	362.9074	10	$Al_2O(NO_3)_4(HCOO)^-$	0	+1	0	+4
377.9605	377.9643	10	$Al(C_8H_5O_4)(NO_3)_3^-$	+2	0	+1	+3
379.8938	379.8976	10	$Al_2O(NO_3)_5^-$	0	0	0	+5
391.9572	391.9591	5	$Al_2(NO_3)_3(OCH_3)_2(HCOO)_2^-$	0	+2	+6	+3
394.9295	394.9336	10	$Al_2(NO_3)_4(OCH_3)(OH)(HCOO)^-$	0	+1	+4	+4
408.9449	408.9493	11	$Al_2(NO_3)_4(OCH_3)_2(HCOO)^-$	0	+1	+6	+4
425.9348	425.9395	11	$Al_2(NO_3)_5(OCH_3)_2^-$	0	0	+6	+5
451.9542	451.9591	11	$Al_2(C_8H_4O_4)(OCH_3)(OH)(NO_3)_3^-$	+2	0	+4	+3
465.9336	465.9384	10	$Al_2(C_8H_4O_4)(NO_3)_3(OH)(HCOO)^-$	+2	+1	+1	+3
482.9234	482.9285	11	$Al_2(C_8H_4O_4)(NO_3)_4(OH)^-$	+2	0	+1	+4
511.9744	511.9802	11	$Al_2(C_8H_5O_4)(NO_3)_3(OCH_3)_2(HCOO)^-$	+2	+1	+7	+3

514.9490	514.9548	11	$Al_2(C_8H_5O_4)(NO_3)_4(OCH_3)(OH)^-$	+2	0	+5	+4
528.9646	528.9704	11	$Al_2(C_8H_5O_4)(NO_3)_4(OCH_3)_2^-$	+2	0	+7	+4
542.9638	542.9653	3	$Al_3(HCOO)_2(OCH_3)_4(NO_3)_4^-$	0	+2	+12	+4
601.9012	601.9085	12	$Al_3O(C_8H_4O_4)(OCH_3)(NO_3)_5^-$	+2	0	+3	+5
615.8815	615.8877	10	$Al_3O(C_8H_4O_4)(HCOO)(NO_3)_5^-$	+2	+1	0	+5
632.8702	632.8779	12	$Al_3O(C_8H_4O_4)(NO_3)_6^-$	+2	0	0	+6
647.9410	647.9139	42	$Al_3(C_8H_4O_4)(NO_3)_5(OCH_3)(OH)(HCOO)^-$	+2	+1	+4	+5
648.9473	648.9707	36	$Al_3(C_8H_5O_4)(HCOO)(NO_3)_4(OCH_3)_3(OH)^-$	+2	+1	+11	+4
661.9214	661.9296	12	$Al_3(C_8H_4O_4)(NO_3)_5(HCOO)(OCH_3)_2^-$	+2	+1	+6	+5
664.8960	664.9041	12	$Al_3(C_8H_4O_4)(NO_3)_6(OH)(OCH_3)^-$	+2	0	+4	+6
672.9048	672.9132	12	$Al_3O(C_8H_4O_4)_2(NO_3)_4^-$	+4	0	0	+4
678.9115	678.9198	12	$Al_3(C_8H_4O_4)(NO_3)_6(OCH_3)_2^-$	+2	0	+6	+6
704.9306	704.9394	12	$Al_3(C_8H_4O_4)_2(NO_3)_4(OCH_3)(OH)^-$	+4	0	+4	+4
718.9461	718.9551	13	$Al_3(C_8H_4O_4)_2(NO_3)_4(OCH_3)_2^-$	+4	0	+6	+4
735.9023	735.9089	9	$Al_3(C_8H_4O_4)_2(NO_3)_5(OH)^-$	+4	0	+1	+5
798.9066	798.9201	17	$Al_4(C_8H_4O_4)(HCOO)(OCH_3)_3(OH)(NO_3)_6^-$	+2	+1	+10	+6
801.8852	801.8946	12	$Al_4(C_8H_4O_4)(NO_3)_7(OH)_2(OCH_3)_2^-$	+2	0	+8	+7
812.9203	812.9357	19	$Al_4(C_8H_4O_4)(HCOO)(OCH_3)_4(NO_3)_6^-$	+2	+1	+12	+6
815.8999	815.9102	13	$Al_4(C_8H_4O_4)(OCH_3)_3(OH)(NO_3)_7^-$	+2	0	+10	+7
823.9087	823.9194	13	$Al_4O(C_8H_4O_4)_2(OCH_3)_2(NO_3)_5^-$	+4	0	+6	+5
829.9149	829.9259	13	$Al_4(C_8H_4O_4)(OCH_3)_4(NO_3)_7^-$	+2	0	+12	+7
838.9306	838.9918	73	$Al_4(C_8H_4O_4)_2(OCH_3)_5(NO_3)_4^-$	+4	0	+15	+4
841.9190	841.9299	13	$Al_4(C_8H_4O_4)_2(OCH_3)_2(OH)_2(NO_3)_5^-$	+4	0	+8	+5
855.9338	855.9456	14	$Al_4(C_8H_4O_4)_2(OCH_3)_3(OH)(NO_3)_5^-$	+4	0	+10	+5
869.9492	869.9612	14	$Al_4(C_8H_4O_4)_2(OCH_3)_4(NO_3)_5^-$	+4	0	+12	+5

Supplementary Table 2. Measured and calculated m/z as well as their assignment in positive mode.

**m/z observed**	**m/z calculated**	**Error, ppm**	**Assigned to**	**Δ m/z in terephthalic -$^{13}C_2$ acid**	**Δ m/z in DMF -d_7**	**Δ m/z in methanol -d_4**	**Δ m/z in ^{15}N aluminum nitate**
119.1155	119.1179	20	$((CH_3)_2NH_2)(C_3H_7NO)^+$	0	+13	+2	0
154.1155	154.1186	20	$((CH_3)_2NH_2)_2(NO_3)^+$	0	+12	+4	+1
262.1665	262.1721	21	$((CH_3)_2NH_2)_3(NO_3)_2^+$	0	+18	+6	+2
266.0874	266.0927	20	$Al(NO_3)(OCH_3)(C_3H_7NO)_2^+$	0	+14	+3	+1
280.0662	280.0719	20	$Al(HCOO)(NO_3)(C_3H_7NO)_2^+$	0	+15	0	+1
297.0563	297.0621	20	$Al(NO_3)_2(C_3H_7NO)_2^+$	0	+14	0	+2
327.0437	327.0403	10	$Al(C_8H_5O_4)(NO_3)(C_3H_7NO)^+$	+2	+7	+1	+1
327.0360	327.0559	61	$Al_2(HCOO)(NO_3)(OCH_3)_3(C_3H_7NO)^+$	0	+8	+9	+1
341.0312	341.0351	11	$Al_2(NO_3)(HCOO)_2(OCH_3)_2(C_3H_7NO)^+$	0	+9	+6	+1
344.0028	344.0097	20	$Al_2(NO_3)_2(OH)(OCH_3)(HCOO)(C_3H_7NO)^+$	0	+8	+4	+2
358.0177	358.0253	21	$Al_2(NO_3)_2(HCOO)(OCH_3)_2(C_3H_7NO)^+$	0	+8	+6	+2
370.2122	370.2256	36	$((CH_3)_2NH_2)_4(NO_3)_3^+$	0	+24	+8	+3
400.0850	400.0931	20	$Al(C_8H_5O_4)(NO_3)(C_3H_7NO)_2^+$	+2	+14	+1	+1
417.0542	417.0624	20	$Al_2(NO_3)_2(OCH_3)(OH)(HCOO)(C_3H_7NO)_2^+$	0	+15	+14	+2
431.0688	431.0781	22	$Al_2(NO_3)_2(OCH_3)_2(HCOO)(C_3H_7NO)_2^+$	0	+15	+6	+2
447.0680	447.0770	20	$Al_2(C_8H_4O_4)(NO_3)(OCH_3)_2(CH_3OH)(C_3H_7NO)^+$	+2	+7	+10	+1
461.0484	461.0563	17	$Al_2(C_8H_5O_4)(NO_3)(OCH_3)_2(HCOO)(C_3H_7NO)^+$	+2	+8	+7	+1
464.0212	464.0308	21	$Al_2(C_8H_4O_4)(NO_3)_2(OH)(CH_3OH)(C_3H_7NO)^+$	+2	+7	+5	+2
478.0299	478.0464	35	$Al_2(C_8H_4O_4)(NO_3)_2(OCH_3)(CH_3OH)(C_3H_7NO)^+$	+2	+7	+7	+2
478.2399	478.2790	82	$((CH_3)_2NH_2)_5(NO_3)_4^+$	0	+30	+10	+4
537.0727	537.0836	20	$Al_2(C_8H_4O_4)(NO_3)_2(OH)(CH_3OH)(C_3H_7NO)_2^+$	+2	+14	+5	+2

551.0882	551.0992	20	$Al_2(C_8H_4O_4)(NO_3)_2(OCH_3)(CH_3OH)(C_3H_7NO)_2^+$	+2	+14	+7	+2
670.0511	670.0427	13	$Al_3(C_8H_4O_4)(HCOO)(NO_3)_3(OCH_3)(OH)(C_3H_7NO)_2^+$	+2	+15	+4	+3
671.0709	671.0995	43	$Al_3(C_8H_5O_4)(HCOO)(NO_3)_2(OCH_3)_3(OH)(C_3H_7NO)_2^+$	+2	+15	+11	+2
684.0348	684.0584	34	$Al_3(C_8H_4O_4)(HCOO)(NO_3)_3(OCH_3)_2(C_3H_7NO)_2^+$	+2	+15	+6	+3
687.0193	687.0329	20	$Al_3(C_8H_4O_4)(NO_3)_4(OCH_3)(OH)(C_3H_7NO)_2^+$	+2	+14	+4	+4
694.3769	694.3860	13	$((CH_3)_2NH_2)_7(NO_3)_6^+$	0	+42	+14	+6
701.0252	701.0486	33	$Al_3(C_8H_4O_4)(NO_3)_4(OCH_3)_2(C_3H_7NO)_2^+$	+2	+14	+6	+4
802.3473	802.4395	115	$((CH_3)_2NH_2)_8(NO_3)_7^+$	0	+48	+16	+7
838.0219	838.0390	20	$Al_4(C_8H_4O_4)(NO_3)_5(OCH_3)_3(OH)(C_3H_7NO)_2^+$	+2	+14	+10	+5
878.0553	878.0744	22	$Al_4(C_8H_4O_4)_2(NO_3)_3(OCH_3)_3(OH)(C_3H_7NO)_2^+$	+4	+14	+10	+3
910.5077	910.4930	16	$((CH_3)_2NH_2)_9(NO_3)_8^+$	0	+54	+18	+8
